# Density Functional Study on Adsorption of NH_3_ and NO_x_ on the γ-Fe_2_O_3_ (111) Surface

**DOI:** 10.3390/molecules28052371

**Published:** 2023-03-04

**Authors:** Wei Huang, Liang Wang, Lu Dong, Hongyun Hu, Dongdong Ren

**Affiliations:** 1 State Key Laboratory of Coal Combustion, School of Energy and Power Engineering, Huazhong University of Science and Technology, Wuhan 430074, China; 2 China Power Hua Chuang (Suzhou) Electricity Technology Research Company Ltd., Suzhou 215125, China; 3 Research Institute, Huazhong University of Science and Technology in Shenzhen, Wuhan 430074, China; 4 School of Environmental and Municipal Engineering, Qingdao University of Technology, Qingdao 266555, China

**Keywords:** selective catalytic reduction, adsorption, DFT, γ-Fe_2_O_3_

## Abstract

γ-Fe_2_O_3_ is considered to be a promising catalyst for the selective catalytic reduction (SCR) of nitrogen oxide (NO_x_). In this study, first-principle calculations based on the density function theory (DFT) were utilized to explore the adsorption mechanism of NH_3_, NO, and other molecules on γ-Fe_2_O_3_, which is identified as a crucial step in the SCR process to eliminate NO_x_ from coal-fired flue gas. The adsorption characteristics of reactants (NH_3_ and NO_x_) and products (N_2_ and H_2_O) at different active sites of the γ-Fe_2_O_3_ (111) surface were investigated. The results show that the NH_3_ was preferably adsorbed on the octahedral Fe site, with the N atom bonding to the octahedral Fe site. Both octahedral and tetrahedral Fe atoms were likely involved in bonding with the N and O atoms during the NO adsorption. The NO tended to be adsorbed on the tetrahedral Fe site though the combination of the N atom and the Fe site. Meanwhile, the simultaneous bonding of N and O atoms with surface sites made the adsorption more stable than that of single atom bonding. The γ-Fe_2_O_3_ (111) surface exhibited a low adsorption energy for N_2_ and H_2_O, suggesting that they could be adsorbed onto the surface but were readily desorbed, thus facilitating the SCR reaction. This work is conducive to reveal the reaction mechanism of SCR on γ-Fe_2_O_3_ and contributes to the development of low-temperature iron-based SCR catalysts.

## 1. Introduction

The SCR (selective catalytic reduction) NOx removal technology originated in the 1950s and has realized industrial operation since the 1970s [1]. Nowadays, SCR technology has become the most important and efficient method for NOx removal from flue gas in the industrial field [2]. The SCR reaction is a process of selective reduction of NOx to non-toxic and harmless N_2_ and H_2_O by reducing agents, including NH_3_, CH_4_, H_2_, and CO, under specific temperature conditions [3,4]. A catalyst is crucial in the SCR process, which determines the layout and catalytic efficiency of the whole reactor. At present, commercial vanadium–titanium catalysts with good NO_x_ removal efficiency and thermal stability are widely used in the SCR process, in which the V_2_O_5_ served as the primary active component. However, the operating temperature of vanadium–titanium catalysts is relatively high (about 330–400 °C), and the V_2_O_5_ is toxic, resulting in secondary pollution [5]. Therefore, the development of low-operating-temperature and high-efficiency SCR catalysts has attracted extensive attention from researchers [6,7].

Iron-based SCR catalysts have attracted an increasing amount of attention owing to their remarkable capability to reduce NOx at lower temperatures [8,9,10,11,12]. As early as 1981, Kato et al. [13,14] prepared Fe_2_O_3_-TiO_2_ catalysts by using meta titanic acid (TiO(OH)_2_·nH_2_O) and ferric sulfate as precursors. The NO_x_ elimination efficiency of Fe_2_O_3_-TiO_2_ catalysts was over 90%, with a good N_2_ selectivity at the temperature range from 350 °C to 450 °C. Liu et al. [15,16,17] prepared iron–titanium composite oxide catalysts (FeTiO_x_) by co-precipitation method with ferric nitrate and titanium sulfate as precursors. The FeTiO_x_ catalyst could remove more than 90% of NO_x_ in flue gas during 250–400 °C and exhibited good resistance to sulfur poisoning and water. Yang et al. [18,19] prepared a Fe-Ti spinel structure catalyst ((Fe_3−χ_Ti_χ_)_1−δ_O_4_) via the co-precipitation method and tested its NO_x_ removal efficiency. The results showed that the Fe/Ti catalyst exhibited excellent NO_x_ removal performance and good resistance to sulfur poisoning and water. Some researchers have reported that Fe_2_O_3_ exhibited a relatively high SCR denitrification activity. Supported Fe_2_O_3_ was prepared by Bai et al. [20]; the NO_x_ purification efficiency of this sample with a “tube in tube” structure was higher than 98% at 200–250 °C. Yao et al. [8,9] investigated the NO_x_ elimination characteristics of γ-Fe_2_O_3_ in a fluidized bed, and the experiment results indicated that γ-Fe_2_O_3_ exhibited an excellent denitrification ability in the temperature range between 200 and 290 °C. Yang et al. [21] confirmed that γ-Fe_2_O_3_ exhibited a better de-NO_x_ performance than α-Fe_2_O_3_ by denitrification experiments. Liang et al. [22] synthesized nano-γ-Fe_2_O_3_ particles for NO_x_ removal and found that more than 85% NO_x_ could be eliminated at 180–330 °C, and the temperature played a positive role in the range of 90–180 °C. Moreover, about 98% NO_x_ could be removed at 240 °C under the aerobic condition. Up to now, there is still much controversy about the mechanism of SCR reaction. Ramis et al. [23,24] and Qi et al. [25] found that the active component in the catalysts responsible for NH_3_ adsorption might be Lewis acid sites, and the adsorption product would be dehydrogenated to form NH_2_ species. The NH_2_ acted as an intermediate species and subsequently reacted with NO to form another intermediate species, and finally N_2_ and H_2_O were produced after the intermediate species decomposition. However, Gilardoni et al. [26,27] inferred that NH_3_ was adsorbed onto the B-acid sites on the V_2_O_5_ catalyst surface primarily to generate ammonium ion based on the DFT calculation. Furthermore, Rethwisch et al. [28] discovered that the active sites on the catalyst surface could adsorb NO, as well, resulting in the formation of complex adsorption products. The method of in situ infrared diffuse reflectance spectroscopy was applied to investigate the SCR reaction involved in the γ-Fe_2_O_3_ catalyst surface. The results indicated that the L-acid and B-acid sites served as the activate sites which dominated the NH_3_ and NO adsorption. NH_3_ was preferentially attracted by the L-acid sites, and NO_x_ underwent an affinity reaction, with the active sites forming complex N-containing species. Therefore, the following conclusion can be drawn that the study of the Fe-based SCR denitrification mechanisms has recently expanded to include research on the NH_3_ SCR reaction on catalysts. However, as a result of the complexity of the real flue gases, the mechanism of heterogeneous catalytic reaction still lacks a convincing explanation. It is known that the catalytic reaction of the catalyst can always be treated as a “black box” as a result of the difficulty of experimental measurement [29]. In order to comprehend the characteristics of the γ-Fe_2_O_3_ catalyst surface and SCR reaction and to identify the active sites responsible for attracting NH_3_/NO_x_, a computational study of the interaction between flue gas, such as NH_3_ and NO, and the γ-Fe_2_O_3_ (111) surface, using DFT calculations, was performed in the present work.

It has been agreed that, in the NH_3_-SCR reaction, the reactants must first be adsorbed onto the catalyst surface and then activated before the reaction. Therefore, there must be two steps during the SCR-NO_x_ eliminating process: the gas molecules must be adsorbed on the catalyst surface, and the reaction must occur between the reactants. The popularly accepted SCR reaction is known as 4NO + 4NH_3_ + O_2_ → 4N_2_ + 6H_2_O. During the process, the adsorption of reactants such as NH_3_, NO, and O_2_, will firstly occur on the catalyst’s surface, and then the oxidation–reduction reaction can take place. Hence, the adsorption of reactants might play a big part in the de-NO_x_ reaction. The formation of N_2_ and H_2_O during the SCR process underscores the significance of investigating the adsorption of these molecules onto the catalyst surface, making it an intriguing and worthwhile research avenue to explore. With the improvement of theoretical models used in computational chemistry, a growing number of researchers are applying computational chemistry to simulate the surface mechanism of SCR catalysts and to develop new low-temperature SCR catalysts [30,31]. By DFT simulation, parameters of different adsorption configurations on different catalysts, such as adsorption energy, bond length, and bond angle, can be obtained. The optimal adsorption mode of NO_x_ on the catalyst can thus be obtained, as well, which can provide guidance for the SCR reaction mechanism. As reported in our previous studies, tentative research has been performed to explore the adsorption characteristics of denitration reactants and products on the γ-Fe_2_O_3_ (001) surface, indicating that the affinity of these gas molecules varied significantly at different sites on the surface [32]. It has been reported that the γ-Fe_2_O_3_ (111) surface is also considered to be a stable and active catalytic surface, which could have a significant impact on the de-NO_x_ process [33]. As far as we know, the adsorption mechanism of the reactants and products in the SCR reaction on the γ-Fe_2_O_3_ (111) surface has rarely been investigated.

In this study, the adsorption of reactants (NH_3_, NO_x_, and O_2_), and products (N_2_ and H_2_O) over γ-Fe_2_O_3_ (111) surface was investigated by using the DFT calculation. The potential active sites on the surface of γ-Fe_2_O_3_ (111) were determined, and the corresponding adsorption energies were calculated. By analyzing the electronic transfer and electronic orbitals interaction between gas molecules and the catalyst surface before and after the SCR reaction, we explored the mechanisms of different gas molecules binding with the γ-Fe_2_O_3_ (111) surface.

## 2. Results

### 2.1. Adsorption of NH_3_ onto γ-Fe_2_O_3_ (111) Surface

NH_3_, as a reducing agent, plays a big part in the SCR reaction. The adsorption of NH_3_ could affect the NO_x_ removal activity over the catalyst. The N atom in NH_3_ molecule contains unbonded lone-pair electrons; thus, NH_3_ can easily lose electrons and be oxidized. Fe_2_O_3_ crystal, with *3d* orbitals that have enough space to accept electrons, tends to adsorb reducing gas molecules, such as NH_3_.

**Figure 1:**
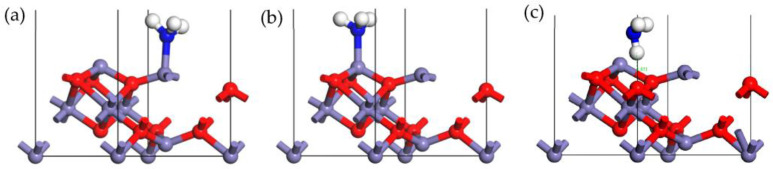
Stable adsorption configurations of NH_3_ on Fe_tet_ site (**a**), Fe_oct_ site (**b**), and Lattice O site (**c**) of the γ-Fe_2_O_3_ (111) surface (N, blue; Fe, purple; H, white; O, red).

The adsorption configurations of the perfect γ-Fe_2_O_3_ (111) surface-adsorbing NH_3_ are shown in Figure 1. The parameters of optimized configurations of the γ-Fe_2_O_3_ (111) surface-adsorbed NH_3_ molecule, such as adsorption energy, Hirshfeld charge, and Bond length, are showed in Table 1. NH_3_ could be bonded to the surface with the N atom and H atom. The octahedral and tetrahedral Fe cations served as the main adsorption sites. Figure 1a,b show that the N atom of NH_3_ was bonded to the tetrahedral Fe site (Fe_tet_) and octahedral Fe site (Fe_oct_), sequentially. Figure 1c shows that the H atom was bonded to the surface via bonding with surface O atom site (Lattice O).

The adsorption energy of configurations (a) and (b) were −8.935 kJ/mol and −90.729 kJ/mol, respectively. It could be found that the octahedral Fe cations were the most active sites, as evidenced by the significantly higher adsorption energy observed in configuration (b), as compared to configuration (a). The Hirshfeld charge of configuration (b) was 0.15 e, suggesting that a large number of electrons were transferred from NH_3_ to the surface atoms. The octahedral Fe cations could attract more electrons than the tetrahedral Fe cations. Hence, the NH_3_ was preferred to be adsorbed by octahedral Fe cations, forming stable bonds. The bond length of Fe-N was 2.130 Å, and the bond angle of H-N-H of NH_3_ was 107.351°, indicating that the bond angle of the NH_3_ molecule was not changed much after adsorption.

The formation of the hydrogen bond was observed between H atom of NH_3_ and lattice oxygen on the surface in configuration (c). The adsorption energy was −34.620 kJ/mol, which was less than that in configuration (b), suggesting that a stable hydrogen bond could be formed between the NH_3_ molecule and γ-Fe_2_O_3_ (111) surface, but it was weaker than the Fe-N bond. By analyzing the Hirshfeld charge, we found that the surface lattice oxygen donated electrons to the NH_3_ molecule, resulting in a net charge of −0.09 e. The bond length of O-H was 1.851 Å, and the bond angle of H-N-H of NH_3_ was 107.949°, indicating that the bond angle of NH_3_ was not changed much after adsorption.

**Figure 2:**
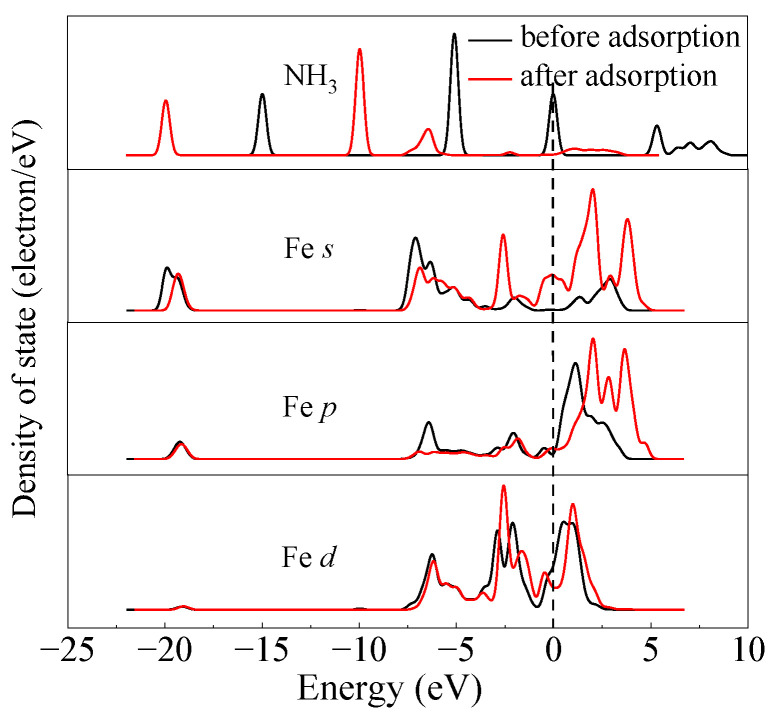
The PDOS for the system before and after NH_3_ adsorption onto the γ-Fe_2_O_3_ (111) surface.

To investigate the mechanism of the Fe-N bond formation, the partial density of state (PDOS) of the NH_3_ and the adjacent Fe atom before and after NH_3_ adsorption were determined. The PDOS of the most stable configuration (b) was determined as exhibited in Figure 2. It can be found that the NH_3_ peak underwent a reduction in intensity and a downward shift in energy level as a result of adsorption, which was consistent with the Hirshfeld charge results that NH_3_ obtains electrons during the adsorption process. An analysis of the NH_3_ and Fe orbitals revealed that the N atom strongly hybridized with the Fe cation with a resonance peak around −7 eV, and this is indicative of the formation of a stable chemical bond. Effective hybridization between the Fe and N atoms is compelling evidence for the formation of a stable chemical bond, specifically the Fe-N bond, which is responsible for the remarkable adsorption strength of NH_3_ on the γ-Fe_2_O_3_ (111) surface.

### 2.2. Adsorption of NO onto γ-Fe_2_O_3_ (111) Surface

**Figure 3:**
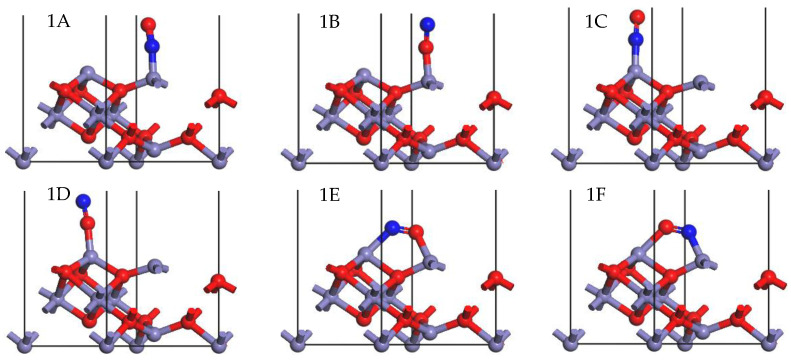
Stable adsorption configurations of NO on the γ-Fe_2_O_3_ (111) surface (N, blue; Fe, purple; O, red).

For the SCR reaction over γ-Fe_2_O_3_, it is crucial for NO to adsorb onto the surface as the first step. The NO adsorption patterns on the γ-Fe_2_O_3_ (111) surface were optimized geometrically, and the resulting parameters can be found in Table 2, with a visual representation in Figure 3. In the configurations 1A, 1B, 1C, and 1D, NO was bonded to the Fe cations with one of its atoms, whereas both N and O atoms were bonded to the surface Fe atoms in the configurations 1E and 1F. The bonding of a nitrogen atom and oxygen atom with tetrahedral Fe cations occurred in configurations 1A and 1B, while configurations 1C and 1D showed bonding between a nitrogen atom and oxygen atom and octahedral Fe cations. In configuration 1E, the N atom bonded with an octahedral Fe cation, while the O atom was linked with a tetrahedral Fe cation. An opposite phenomenon was observed in configuration 1F, as the nitrogen atom and oxygen atom were combined with the tetrahedral Fe cation and the octahedral Fe cation, respectively.

In the configurations 1A–1D, the adsorption energy follows 1A > 1B > 1C > 1D. It can be seen that the N atom preferred to be adsorbed onto both of the two Fe cations to form a stable configuration, which is known as the nitroso acyl compound [34]. When NO was close to the surface, it attracted electrons due to its oxidizability, as the Hirshfeld charge change of NO was negative in these four configurations. The higher electronegativity of the O atom in configurations 1B and 1D resulted in a stronger electron attraction compared to the N atom. The length of the Fe-O bond was longer than that of the Fe-N bond because the N atom radio was smaller than the O atom radio, which could explain the fact that the Fe-N was more stable. In addition, compared with octahedral Fe, NO would attract more electrons when it was adsorbed on the tetrahedral Fe. Thus, more electrons will enter the bonding orbital and form a more stable chemical bond, resulting in a shorter Fe-N bond.

**Figure 4:**
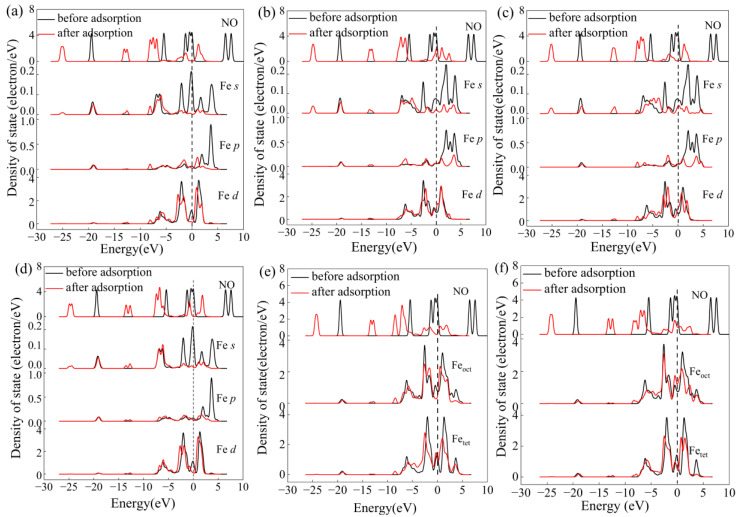
The PDOS for the system before and after NO adsorption onto the γ-Fe_2_O_3_ (111) surface. PDOS of configuration 1A (**a**), PDOS of configuration 1B (**b**), PDOS of configuration 1C (**c**), PDOS of configuration 1D (**d**), PDOS of configuration 1E (**e**), PDOS of configuration 1F (**f**).

For the purpose of explaining the mechanism of bond formation, the PDOS for configurations 1A–1F was also analyzed, as exhibited in Figure 4. After NO adsorption, it is evident from Figure 4a that the NO molecule orbitals were moved to a less energetic state. Hybrid peaks were observed between the NO orbital and the Fe atom’s *p* and *d* orbitals at approximately −6 eV to −8 eV, indicating the formation of a stable adsorption configuration. The same results could be found in configuration 1C. However, compared with Figure 4a, the hybrid peaks were weaker, which could be attributed to the lower adsorption energy of configuration 1C. It could thus be concluded that NO preferred to be adsorbed onto the tetrahedral Fe cation. After adsorption onto configuration 1B, the orbitals of the NO molecule underwent a shift to a less energetic state. The orbital of NO was hybridized with the *p* and *d* orbitals of Fe atom at approximately −6 eV~−7 eV. The hybrid peaks were lower in configuration 1B when compared with configuration 1A, suggesting that the Fe-N bond was more stable than the Fe-O bond.

In configurations 1E and 1F, there were two bonds formed. Thus, their adsorption was more stable than that of the configurations 1A and 1B. The adsorption energy of configuration 1F was higher than that of configuration 1E due to the bond between the N atom and tetrahedral Fe being stronger in configuration 1F. As indicated in the PDOS (shown in Figure 4e,f), the orbital of NO atom shift downward, its energy was lowered, and the appearance of hybrid peaks around −6 eV to −8 eV suggested the formation of a chemical bond between the NO and Fe site.

### 2.3. Adsorption of NO_2_ onto γ-Fe_2_O_3_ (111) Surface

During the SCR process, NO could react with O_2_ to produce NO_2_, which could also be adsorbed onto the catalyst’s surface, and then participated in the reaction. Hence, it is also necessary to study the adsorption of NO_2_ over γ-Fe_2_O_3_.

**Figure 5:**
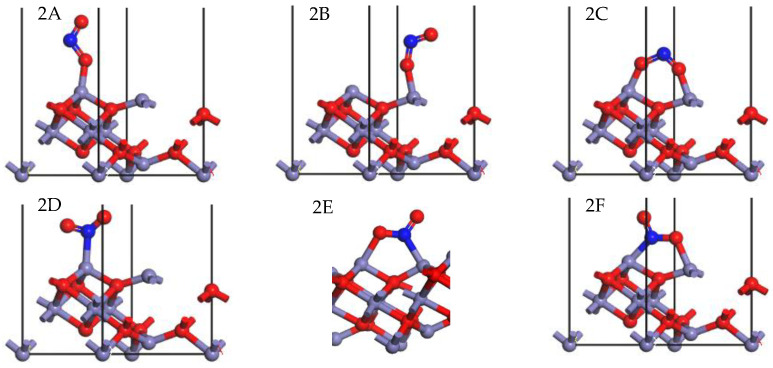
Stable adsorption configurations of NO_2_ onto the γ-Fe_2_O_3_ (111) surface (N, blue; Fe, purple; O, red).

All possible orientations (parallel and perpendicular) and adsorption sites were taken into consideration for the NO_2_ molecule adsorbed onto the γ-Fe_2_O_3_ (111) surface. The optimized and stable configurations are demonstrated in Figure 5. It can be seen that configurations 2A and 2B are linear configurations, in which the NO_2_ molecule bonded to the surface through one of its O atoms, which was linked to the octahedral Fe and tetrahedral Fe cations separately, and this can be identified as monodentate nitrite [34]. The adsorption energy of configurations 2A and 2B was −250.395 kJ/mol and −240.995 kJ/mol, respectively. NO_2_ preferred to be adsorbed onto the octahedral Fe cation in linear configurations, but there was not much difference between configurations 2A and 2B. In configuration 2C, both of the two O atoms in the NO_2_ molecule were connected with the octahedral Fe and tetrahedral Fe cations of the γ-Fe_2_O_3_ simultaneously, and this configuration was named bridging bidentate nitrite [29]. The adsorption energy of configuration 2C was −295.858 kJ/mol, which was higher than that of configurations 2A and 2B because two bonds were formed.

In configuration (d), the N atom formed a bond with the octahedral Fe site, with an adsorption energy of −262.002 kJ/mol, which was higher than that of configurations 2A and 2B but still lower than that of configuration 2C. It could be concluded that the Fe-N bond was easily formed, and it was more stable compared with the Fe-O bond. The N-containing compound formed in configuration 2D could be ascribed to the nitro compound [22]. As for configurations 2E and 2F, the N atom and one of the O atoms formed bonds with two Fe atoms, and these kinds of configuration are known as chelating nitro compounds [34]. In configuration 2E, the O atom was bonded to the octahedral Fe cation, and the N atom was bonded to the tetrahedral Fe cation. In configuration 2F, however, the O atom formed a bond with the tetrahedral Fe site, while the N atom was bonded to the octahedral Fe cation. The adsorption energy of configurations 2E and 2F were −329.365 kJ/mol and −345.194 kJ/mol, respectively, which were much higher than those of configurations 2A and 2B. This can be attributed to the fact that there were two bonds formed, in which one bond was the stable Fe-N bond and the other one was the Fe-O bond. The adsorption energy of configuration 2F was a little higher than that of 2E, and this indicated that the octahedral Fe cation had a higher activity to bond to the N atom and formed a stable configuration.

**Figure 6:**
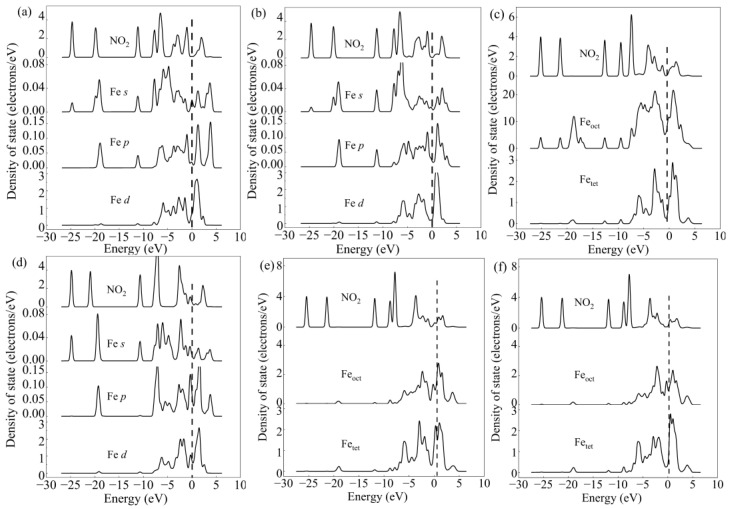
The PDOS for the system before and after NO_2_ adsorption on the γ-Fe_2_O_3_ (111) surface. PDOS of configuration 2A (**a**), PDOS of configuration 2B (**b**), PDOS of configuration 2C (**c**), PDOS of configuration 2D (**d**), PDOS of configuration 2E (**e**), PDOS of configuration 2F (**f**).

To further understand the mechanism of NO_2_ adsorption, the PDOS of the corresponding configurations after adsorption (listed in Figure 5) were analyzed, as presented in Figure 6.

In configurations 2A and 2B, the O atom bonded with the Fe cation, and it can be seen that the orbital of the NO_2_ molecule hybridized with the adjacent Fe atoms at about −7.5 eV and −2.5 eV after adsorption, resulting in a stable chemical bond between NO_2_ and the catalyst’s surface. In configuration 2C, hybrid peaks between NO_2_ and both tetrahedral and octahedral Fe sites appeared at −7.5 eV and −2.0 eV, suggesting that the chemical bond was formed between two kinds of Fe cations and NO_2_ molecules. In configuration 2D, the hybrid peak was also formed because the nitrogen atom of NO_2_ could also form a stable chemical bond with the Fe atom. As seen from configurations 2E and 2F, both of the N and O atoms were connected with the adjacent Fe atoms simultaneously, and the hybrid peak between the NO_2_ molecule and tetrahedral and octahedral Fe cations also appeared. It can be seen that NO_2_ could be attracted by the γ-Fe_2_O_3_ (111) surface, with both N and O atoms bonding to the Fe cation and forming a stable chemical bond.

To sum up, the adsorption of NO_x_, including NO and NO_2_, can be completed on the γ-Fe_2_O_3_ (111) surface. Both NO and NO_2_ could be attracted by the surface’s active sites, and the N atom and O atom could both bond to the Fe cation on the surface since the orbital of the molecule could be hybridized with the iron oxide surface. In the NO_x_ adsorption process, a number of electrons were transferred from the catalyst’s surface to the gas species because of the oxidizability of NO_x_. The adsorbed NO_x_ could be formed as the N-related species, such as nitrate, bidentate nitrate, monodentate nitrate, and monodentate nitrite, on the γ-Fe_2_O_3_ (111) surface, and this was in accordance with the in situ infrared results in the previous study [22]. According to the analysis of NO_x_ and NH_3_ adsorption, it can be deduced that the L-H reaction mechanism is suitable for describing the SCR reaction process [35], suggesting that the simultaneous adsorption of ammonia and NO on the γ-Fe_2_O_3_ (111) surface existed during the de-NO_x_ reaction. Thus, the SCR denitration process over the γ-Fe_2_O_3_ catalyst can easily occur under a lower temperature [27].

### 2.4. Adsorption of Other Molecules onto the γ-Fe_2_O_3_ (111) Surface

**Figure 7:**
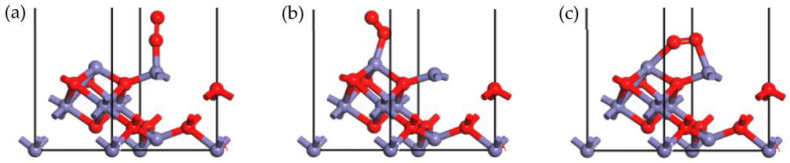
Stable adsorption configurations of O_2_ onto Fe_tet_ site (**a**), Fe_oct_ site (**b**), and Fe_tet_ site + Fe_oct_ site (**c**) of the γ-Fe_2_O_3_ (111) surface (N, blue; Fe, purple; O, red).

In the SCR process, O_2_, an important reactant, plays an important role, which could promote the NO_x_ removal activity. Studying the adsorption of O_2_ is conducive to understanding the entire SCR reaction. The configurations of the O_2_ adsorption onto the γ-Fe_2_O_3_ (111) surface were optimized as shown in Figure 7.

Figure 7a depicts the stable configuration in which O_2_ adsorbed onto the tetrahedral Fe cation, and Figure 7b depicts the stable adsorption of O_2_ on the octahedral Fe atom. Meanwhile, in the Figure 7c, the two O atoms of the O_2_ molecule were connected with the tetrahedral and octahedral Fe atoms simultaneously. The adsorption energy of the configurations in Figure 7a–c is −133.470 kJ/mol, −174.754 kJ/mol, and −285.242 kJ/mol, respectively. The adsorption energy of the configuration in Figure 7c is much higher than that of configurations in Figure 7a,b because two chemical bonds were formed in this configuration, as listed in Figure 7c. The adsorption energy of the configuration in Figure 7b was higher than that of configuration in Figure 7a, thus indicating that the octahedral Fe sites were more active than tetrahedral Fe cations during the O_2_ adsorption process. The Hirshfeld charge analysis showed that the O_2_ molecule could obtain a lot of electrons from the surface atoms. The Hirshfeld charge calculated in these three configurations was −0.27, −0.25, and −0.32, respectively. It is easy to know that the adsorption mechanism of O_2_ was identical to that of NO since they were both diatomic molecules. During the process of O_2_ adsorption, O_2_ acquired a number of electrons from the γ-Fe_2_O_3_ (111) surface, and a stable chemical bond was formed. The results showed that energy was released during the adsorption process, indicating that O_2_ could be easily adsorbed onto the γ-Fe_2_O_3_ (111) surface and that the γ-Fe_2_O_3_ (111) surface should exhibit good NO_x_ removal activity.

According to the SCR reaction, N_2_ and H_2_O will be generated on the catalyst surface, and their desorption behavior would affect the SCR process. Thus, it is important to study the desorption of N_2_ and H_2_O.

**Figure 8:**
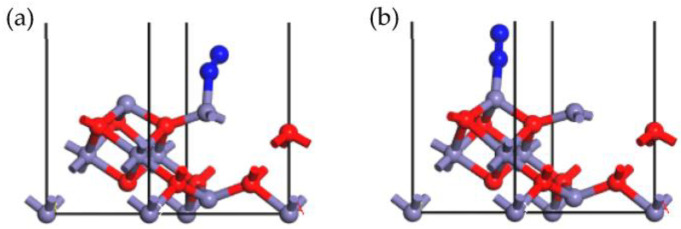
Stable adsorption configurations of N_2_ onto Fe_tet_ site (**a**), Fe_oct_ site (**b**) of the γ-Fe_2_O_3_ (111) surface (N, blue; Fe, purple; O, red).

The N_2_ adsorption patterns on the γ-Fe_2_O_3_ (111) surface were optimized as illustrated in Figure 8, and the resulting parameters can be found in Table 3. The stable adsorption configurations in Figure 8a,b showed that N_2_ was adsorbed onto the tetrahedral Fe and octahedral Fe cations, respectively. It could be seen that the Fe cation was the active site for the N_2_ adsorption process, and the N atom could be bonded with the Fe cation to form a stable chemical bond, such as O_2_ and NO. The configuration in Figure 8b obtained a much higher adsorption energy (−123.374 kJ/mol) than obtained in Figure 8a, with an adsorption energy of −86.538 kJ/mol, indicating that the octahedral Fe site was more active than the tetrahedral Fe site in relation to N_2_ adsorption. The Hirshfeld charge of N_2_ in both configurations was negative, suggesting that electrons were transferred from the catalyst to the N_2_ atom since the oxidation of N_2_ occurred. However, compared with the adsorption of NO and O_2_, we observed that fewer electrons were transferred from N_2_, and this could be ascribed to the lower oxidizability of the N_2_ molecule. Therefore, the chemical bond formed during the N_2_ adsorption onto the γ-Fe_2_O_3_ (111) surface was weaker, and the adsorption energy was lower, indicating that N_2_ could not easily be adsorbed compared with O_2_ and NO, even though their molecular structures were very similar. In other words, N_2_, as a product in the SCR process, could be easily desorbed from the γ-Fe_2_O_3_ (111) surface, hence contributing to the positive progress of the SCR reaction.

**Figure 9:**
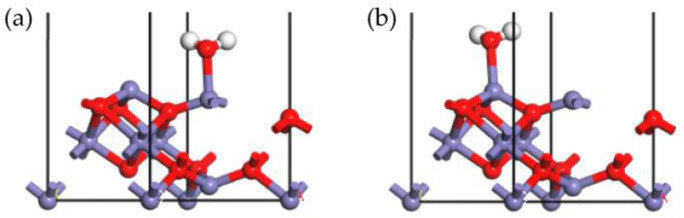
Stable adsorption configurations of H_2_O onto Fe_tet_ site (**a**), Fe_oct_ site (**b**) of the γ-Fe_2_O_3_ (111) surface (Fe, purple; H, white; O, red).

The adsorption configuration of H_2_O on the γ-Fe_2_O_3_ (111) surface was also optimized, as exhibited in Figure 9. The adsorption parameters of these two configurations are shown in Table 4. It is obvious that the O atom of H_2_O was bonded with the Fe cation on the γ-Fe_2_O_3_ (111) surface; the configuration in Figure 9a was H_2_O adsorbed onto the tetrahedral Fe, and configuration in Figure 9b was H_2_O adsorbed onto the octahedral Fe cation. Unlike other molecules, a H_2_O molecule is a nonlinear molecule composed of three atoms. Among the three atoms, the O atoms isolated pairs of electrons, making it easy to lose electrons in the process of adsorption, exhibiting strong reducibility, and showing similar characteristics to ammonia molecule. The Hirshfeld charge results also verified that only a few electrons were transferred from H_2_O to the γ- Fe_2_O_3_ (111) surface in both configurations. The adsorption energy of configurations (a) and (b) were −75.266 kJ/mol and −112.224 kJ/mol, respectively, indicating that H_2_O could be easily desorbed from the γ-Fe_2_O_3_ (111) surface, and this was also beneficial to the positive progress of the SCR reaction.

## 3. Discussion

It has been verified that both the (001) crystal surface and (111) crystal surface are exposed to the surface of γ-Fe_2_O_3_ at the same time, they are the main crystal surfaces of the γ-Fe_2_O_3_ catalyst [22]. During the SCR reaction, the relevant gas molecules can be adsorbed and subsequently be reacted on these two surfaces, so both of these two crystal surfaces need to be investigated. The adsorption of gas molecules participating in NH_3_-SCR reaction on the γ-Fe_2_O_3_ (0011) surface has been analyzed in detail in our previous research [32]. Hence, in this study, the adsorption of the reactants (NH_3_, NO_x_, and O_2_), and the products (N_2_ and H_2_O) over the γ-Fe_2_O_3_ (111) surface was investigated.

On the surface of γ-Fe_2_O_3_ (111), the stable adsorption configurations and adsorption energy were determined. Possible binding mechanisms of various gas molecules on γ-Fe_2_O_3_ (111) surface after the adsorption reaction were also deduced. As compared with results of the NH_3_ and NO_x_ molecules adsorbing on the γ-Fe_2_O_3_ (001) surface [32], the adsorption energies of N_2_ and H_2_O adsorbed on the γ-Fe_2_O_3_ (111) surface were smaller than those adsorbed on the γ-Fe_2_O_3_ (001) surface, which indicated that the products N_2_ and H_2_O generated after NH_3_-SCR reaction were easier to desorption. This means that the SCR reaction on the γ-Fe_2_O_3_ (111) surface should occur more easily.

**Figure 10:**
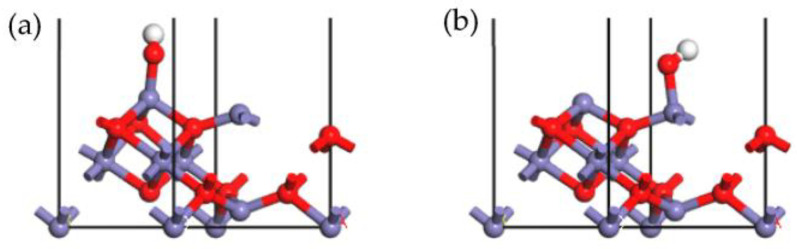
Stable adsorption configurations of -OH onto Fe_oct_ site (**a**), Fe_tet_ site (**b**) of the γ-Fe_2_O_3_ (111) surface (Fe, purple; H, white; O, red).

It should be noted that, after cutting the surface of the γ-Fe_2_O_3_ crystal, the surface Fe and O atoms have the possibility of adsorbing hydrogen or hydroxyl groups. It is undeniable that the hydrogen atom or hydroxyl functional group on the surface will affect the adsorption of NO_x_, NH_3_, and other gas molecules. In this work, the pre-adsorption of hydroxyl on octahedral Fe and tetrahedral Fe sites was calculated, and its effect on NH_3_, NO_x_, and O_2_ adsorbing onto the γ-Fe_2_O_3_ (111) surface was studied. The hydroxyl group was first placed on the γ-Fe_2_O_3_ (111) surface, with the oxygen atom close to the octahedral Fe and tetrahedral Fe atoms perpendicular to the surface, and then optimized calculations were performed. The stable configurations are obtained as illustrated in Figure 10.

**Figure 11:**
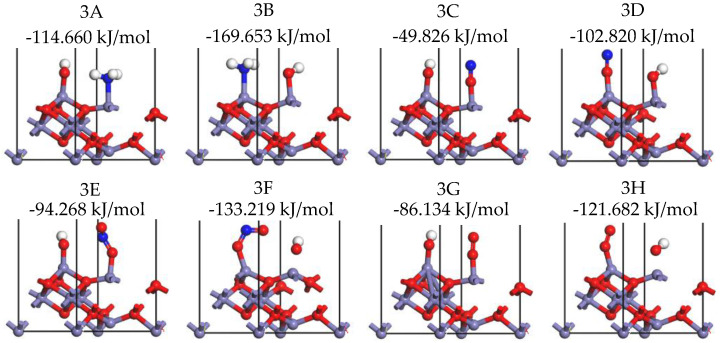
Stable adsorption configurations of NH_3_ on the -OH pre-adsorbed γ-Fe_2_O_3_ (111) surface (N, blue; Fe, purple; H, white; O, red).

After the adsorption of -OH, the NH_3_, NO, NO_2_, and O_2_ gas molecules were placed above the other iron atom site on the γ-Fe_2_O_3_ (111) surface that was not occupied by -OH. The stable adsorption configurations (3A–3H) are presented in Figure 11, and the adsorption energy of the corresponding configurations are also listed. It can be seen that the pre-adsorption of -OH had an obvious promoting effect on the adsorption of NH_3_ (configurations 3A and 3B), and the adsorption energy increased from 8.935 and 90.729 kJ/mol of unabsorbed -OH cases to 114.660 and 169.653 kJ/mol of pre-adsorbed -OH cases. For the adsorption of NO (configurations 3C and 3D), NO_2_ (configurations 3E and 3F), and O_2_ (configurations 3G and 3H), -OH pre-adsorption has a certain inhibitory effect on the adsorption of these molecules. In particular, the adsorption energy of NO_2_ and O_2_ decreased significantly. Therefore, it can be found that the adsorption of hydroxyl groups on the surface has a complex effect on the adsorption of gas molecules participating in the NH_3_-SCR reaction, and this needs to be further considered in future research.

## 4. Materials and Methods

**Figure 12:**
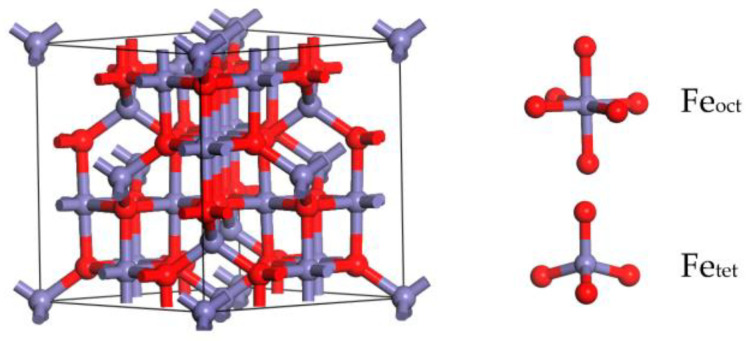
The unit cell of γ-Fe_2_O_3_, showing the Fe cations (in purple) and oxygen ions (in red).

The γ-Fe_2_O_3_ is generally accepted as a maghemite, as it possesses a spinel-type structure, which bears a striking resemblance to magnetite. The crystal structure of the γ-Fe_2_O_3_ unit cell is exhibited in Figure 12, which can be formulated as Fe_21.33_O_32_, with a space group of *Fd-3m* [29,36]. The cubic unit cell has a lattice constant of 0.834 nm and contains 32 O^2−^ anions and 21^1/^_3_ Fe^3+^ cations. The Fe cations associated with the octahedral sites are regarded as Fe_oct_, and the Fe cations associated with the tetrahedral sites are regarded as Fe_tet_. Eight Fe^3+^ cations are located at eight tetrahedral sites, and an additional 13^1/^_3_ Fe^3+^ cations are located among sixteen octahedral sites. In addition, every O site in the crystal is occupied [32].

**Figure 13:**
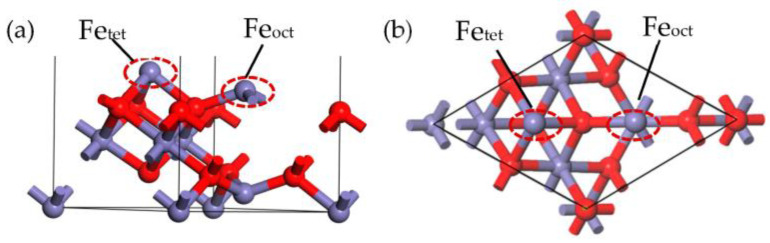
The side view of γ-Fe_2_O_3_ (111) (**a**) and the top view of γ-Fe_2_O_3_ (111) surface (**b**) (N, blue; Fe, purple; O, red).

The γ-Fe_2_O_3_ (001) and γ-Fe_2_O_3_ (111) surfaces are the most widespread surfaces that exist in the γ-Fe_2_O_3_ catalyst. During the SCR reaction, these two surfaces could determine the SCR efficiency, the reactants (NH_3_, NO_x_, and O_2_), and the products (N_2_, H_2_O) that could be adsorbed on these two surfaces. However, there are few studies about the adsorption of NH_3_/NO_x_ on the γ-Fe_2_O_3_ (111) surface. In our previous studies, the γ-Fe_2_O_3_ (001) surface was investigated in detail for the adsorption behaviors of SCR reactants [32,37]. In the present work, the unit cell was firstly optimized, and then, after that, the (111) crystal surface of the γ-Fe_2_O_3_ with Fe_oct_ and Fe_tet_ active sites was identified and treated as the adsorption surface [38]. A p (1 × 1) 7-layer γ-Fe_2_O_3_ (111) slab model was built and separated by a vacuum layer of 20 Å to eliminate the interactions between slabs, as shown in Figure 13.

All spin-polarized DFT calculations were performed using the CASTEP (Cambridge Sequential Total Energy Package) program [39]. The generalized gradient approximation (GGA) with the Perdew–Burke–Ernzerhof (PBE) functional was adopted to estimate the exchange–correlation effects [40]. Ionic cores were described by the ultra-soft pseudopotential method. The electronic wave function was expanded in a plane wave basis set with an energy cutoff of 300 eV. The k-point of the Brillouin zone was 4 × 4 × 1 for all calculations [41]. During the calculation, the first 3 layers were totally relaxed, and the remaining layers were fixed for all the structure optimization and energy calculations.

Adsorption energy is generally considered to be a key criterion for evaluating the adsorption ability. A larger adsorption energy indicates a more stable adsorption. The adsorption energy (*E*_ads_) of different molecules on the γ-Fe_2_O_3_ (111) surface was calculated based on the following expression:

*E*_ads_ = *E*_(surface+molecule)_ − (*E*_surface_ + *E*_molecule_)

where *E*_(surface+molecule)_, *E*_surface_, and *E*_molecule_ are the total energies of the γ-Fe_2_O_3_ (111) surface plus molecule surface system, the pristine γ-Fe_2_O_3_ (111) surface, and gas phase molecule, respectively. The reactants (NH_3_, NO_x_, and O_2_) and products (N_2_ and H_2_O) were optimized in a 10 Å × 10 Å × 10 Å cell to obtain the corresponding energy (*E*_molecule_). The PDOS and the Hirshfeld charge distribution of different adsorption configurations were performed to uncover the bonding mechanisms.

## 5. Conclusions

The present work focused on the adsorption mechanism of reactants (NH_3_, NO_x_, and O_2_) and products (N_2_ and H_2_O) over the γ-Fe_2_O_3_ (111) surface, which is of great significance for the catalytic oxidation of NO. DFT calculations were performed to investigate the adsorption characteristics of these molecules on the γ-Fe_2_O_3_ (111) surface. The results indicated that NH_3_, NO, and NO_2_ can be effectively adsorbed on the γ-Fe_2_O_3_ (111) surface, which is conducive to the de-NO_x_ process. Two kinds of Fe cations, Fe_tet_ cation and Fe_oct_ cation, existed on the surface, and both of them exhibited a certain reactivity in the case of the adsorption of gas molecules participating in the SCR reaction.

During the NH_3_ adsorption process, the octahedral Fe site is the most active site, and the N atom has the ability to bind with the two Fe cations to form a stable chemical bond, as demonstrated by the fact that the N atom was strongly hybridized with Fe cation. During the NO adsorption process, both N and O atoms could be bonded to Fe_tet_ and Fe_oct_ cations. In this case, the N atom exhibits a more stable bond interaction with surface Fe sites than the O atom, and several N-containing species, such as nitrate, bidentate nitrate, etc., are formed. O_2_ could also establish stable bonds with surface Fe cations by transferring electrons from the iron oxide surface to O_2_ in three configurations. The adsorption energy of N_2_ and H_2_O on γ-Fe_2_O_3_ (111) is inferior to that of NH_3_ and NO_x_ on γ-Fe_2_O_3_ (111), which enables H_2_O and N_2_, as products, to be easily desorbed from the surface, thus contributing to the positive progress of the SCR reaction.

In addition, the impact of the pre-adsorbed hydroxyl group on the adsorption of molecules such as NH_3,_ NO, NO_2_, and O_2_ on the γ-Fe_2_O_3_ (111) surface was also investigated. It was found that -OH can be stably bonded to surface Fe cations, which was beneficial to the adsorption of NH_3_. However, the adsorption of NO, NO_2_, and O_2_ molecules was inhibited by -OH to a certain extent. The impact of -OH on the SCR reaction in relation to the γ-Fe_2_O_3_ (111) surface would be an interesting research topic in the future.

## Figures and Tables

**Table 1:** The parameters of optimized configurations of the γ-Fe_2_O_3_ (111) surface-adsorbed NH_3_ molecule.

	Adsorption Site	Adsorption Energy (kJ/mol)	Hirshfeld Charge (e)	Bond Length (Å)
a	Fe_oct_	−8.935	0.10	2.101
b	Fe_tet_	−90.729	0.15	2.130
c	Lattice O	−34.620	−0.09	1.851

**Table 2:** The parameters of optimized configurations of the γ-Fe_2_O_3_ (111) surface-adsorbed NO molecule.

	Adsorption Site	Adsorption Energy (kJ/mol)	Hirshfeld Charge (e)	Fe-N Bond Length (Å)	Fe-O Bond Length (Å)
1A	Fe_oct_	−287.159	−0.14	1.697	
1B	Fe_oct_	−177.547	−0.18		1.811
1C	Fe_tet_	−195.665	−0.10	1.701	
1D	Fe_tet_	−70.593	−0.14		1.802
1E	Fe_tet_-Fe_oct_	−237.602	−0.18	1.941	1.851
1D	Fe_tet_-Fe_oct_	−316.855	−0.16	1.718	2.002

**Table 3:** The parameters of optimized configurations of the γ-Fe_2_O_3_ (111) surface-adsorbed N_2_ molecule.

	Adsorption Site	Adsorption Energy (kJ/mol)	Hirshfeld Charge (e)
a	Fe_oct_	−86.538	−0.10
b	Fe_tet_	−123.374	−0.08

**Table 4:** The parameters of optimized configurations of the γ-Fe_2_O_3_ (111) surface-adsorbed H_2_O molecule.

	Adsorption Site	Adsorption Energy (kJ/mol)	Hirshfeld Charge (e)
a	Fe_oct_	−75.266	0.09
b	Fe_tet_	−112.224	0.11

## Data Availability

Data are available upon request.
